# Public Perceptions and Career Intentions Regarding Clinical Nurse Specialist and Physician Associate Roles: A Cross‐Sectional Survey

**DOI:** 10.1111/inr.70145

**Published:** 2025-12-31

**Authors:** Merav Ben Natan, Yuval Ben Zeev, Nicole Bezelanski

**Affiliations:** ^1^ Department of Nursing Tel Aviv University Tel Aviv Israel; ^2^ Academic School of Nursing, Hillel Yaffe Medical Center Hadera Israel

**Keywords:** advanced clinical roles, career motivation, clinical nurse specialist, gender, health workforce, healthcare education, physician associate, public perception

## Abstract

**Aim:**

To assess public awareness, perceptions, and educational intentions regarding Clinical Nurse Specialist (CNS) and Physician Associate (PA) roles in Israel, and to identify predictors of interest in these career pathways.

**Background:**

Amid physician shortages, countries are promoting advanced roles such as CNSs and PAs to strengthen healthcare capacity. In Israel, both roles are relatively new and underrecognized.

**Methods:**

A cross‐sectional survey of 200 adults in Israel used descriptive statistics to assess familiarity and interest in CNS and PA roles. Two multiple linear regression models identified predictors of interest in pursuing each role's educational pathway.

**Findings:**

Awareness of advanced roles was limited: 25.5% and 21% reported familiarity with CNSs and PAs, respectively. Despite this, 26.5% expressed interest in the CNS pathway and 17% in the PA pathway. Women were more likely to favor the CNS role; men showed greater interest in the PA role. Self‐efficacy, career motivation, and gender predicted CNS interest (*R* ^2^ = 0.332). PA interest was predicted by academic background and gender (*R* ^2^ = 0.256), but not by self‐efficacy or motivation.

**Discussion:**

These findings highlight the need to clarify role definitions, address gendered perceptions, and implement targeted outreach. Differences in predictors suggest that CNS and PA roles appeal to distinct motivational and cognitive profiles.

**Conclusion:**

Despite low awareness, there is substantial public interest in CNS and PA roles. Tailored educational and communication strategies are needed to increase understanding and engagement.

**Implications for Nursing:**

Nurses should use clinical encounters to explain CNS and PA roles and build trust.

**Implications for Health Policy:**

Policymakers should support awareness campaigns, training incentives, and curricular integration to promote role legitimacy and address global workforce challenges.

## 1 Introduction

Health systems worldwide face growing pressures due to physician shortages, increasing demand for accessible and high‐quality care, and the complexity of modern patient needs (Treister‐Goltzman and Peleg 2023). In response, policymakers introduced advanced practice roles, such as Clinical Nurse Specialists (CNSs) and Physician Associates (PAs), to alleviate workforce burdens and improve the delivery of healthcare services (Bourke et al. 2024; Maoz‐Breuer et al. 2019; McKee et al. 2025). These roles, which originated in North America, extended clinical responsibilities beyond the traditional physician‐led model and contributed to greater healthcare system efficiency (Hooker et al. 2017; Ryder et al. 2020).

CNSs were defined as registered nurses with advanced academic qualifications, typically at the master's level or higher, and clinical specialization in fields such as critical care, oncology, or geriatrics (Gullick et al. 2019). These practitioners provided expert care, led quality improvement initiatives, consulted across multidisciplinary teams, and contributed to education and research. Evidence showed that CNSs improved patient outcomes, shortened hospital stays, and supported evidence‐based practice implementation (Latter et al. 2019; Moote et al. 2019).

In Israel, the introduction of CNS and PA roles remains limited, and the number of professionals choosing these academic pathways is still small compared with international benchmarks. As of 2023, only about 683 CNSs and approximately 100 certified PAs were practicing nationwide (Ministry of Health 2024; Nursing Administration 2023). This low uptake reflects not only the novelty of these roles but also the lack of public familiarity with their educational and professional scope. While policy frameworks have endorsed their integration (Maier and Aiken 2016; Treister‐Goltzman and Peleg 2023), public engagement and awareness remain insufficient to support large‐scale adoption.

The rationale for this study stems from the need to understand the factors underlying the persistently low awareness and recruitment to CNS and PA pathways, despite ongoing national efforts to address workforce shortages. Previous research in Israel focused mainly on institutional or professional perspectives (Dickman et al. 2018; Maoz‐Breuer et al. 2019) but did not examine how motivational and cognitive factors influence the general public's interest in pursuing these roles. The absence of such data limits the ability of policymakers and educators to design effective recruitment strategies.

This study examined self‐efficacy, career motivation, intrinsic interest, and perceived knowledge as predictors, drawing on Social Cognitive Career Theory (Lent and Brown 2019) and prior evidence on healthcare career choice. Studies show that perceived capability and motivation are key determinants of career decision‐making in health professions (Gullick et al. 2019; Rizzolo et al. 2017). For example, it was found that nursing students with higher self‐efficacy were more likely to pursue advanced practice roles (Gullick et al. 2019), whereas intrinsic motivation and perceived role clarity were shown to influence interest in PA careers (Cawley and Hooker 2018; Rizzolo et al. 2017). Therefore, examining these predictors within the Israeli public can clarify the underlying factors shaping interest in emerging healthcare roles.

To date, limited evidence exists regarding how policymakers and health authorities communicate about CNS and PA roles in Israel. National strategies have focused primarily on internal workforce planning rather than public information campaigns (Nursing Administration 2023; Ministry of Health 2024). Internationally, efforts such as media campaigns, community programs, and collaborations with educational institutions have helped increase public awareness of advanced practice roles (Swainston et al. 2024; Zhao et al. 2024). Understanding the Israeli public's perceptions and motivational drivers can therefore inform similar communication strategies and guide future policy interventions. Building upon this context, the present study was designed to provide empirical evidence addressing these gaps and to guide future educational and policy efforts related to advanced clinical roles in Israel.

### 1.1 Study Objectives

The primary objective of this study was to investigate public perceptions, perceived knowledge, and educational intentions regarding CNS and PA, which are two emerging advanced clinical roles in the Israeli healthcare system. The secondary objective was to identify motivational, cognitive, and demographic factors associated with public interest in pursuing CNS and PA educational pathways. Specifically, the study examined how self‐efficacy, career motivation, intrinsic interest, perceived knowledge, and gender influence educational intentions. As the first empirical investigation of its kind in Israel, this study contributes evidence that can guide national policies, workforce planning, and educational initiatives aimed at promoting awareness and recruitment to advanced clinical roles.

## 2 Methods

### 2.1 Study Design

This study employed a cross‐sectional, correlational design.

### 2.2 Participants

The study sample consisted of 200 Hebrew‐speaking adults residing in Israel who expressed interest in pursuing academic or professional training. Recruitment was conducted via social media platforms, specifically within Facebook groups related to academic study and higher education in general, not limited to health‐related professions. A convenience sampling strategy was used to reach individuals who were readily accessible and willing to participate. Inclusion criteria were adults aged 18 years and older who are Hebrew speakers and who expressed interest in exploring or pursuing academic studies. Participants were not required to have a preexisting intention to pursue a healthcare career, as the study aimed to assess public perceptions, awareness, and potential interest in emerging clinical roles (CNS and PA) among the general adult population, not only among those already considering healthcare professions. Exclusion criteria included individuals under the age of 18, those who were not proficient in Hebrew, or those who indicated no interest in any form of academic or professional training.

Sample size was determined using G*Power 3.1 to detect a small‐to‐medium effect size (f^2^ = 0.08) with 80% power and a 5% significance level in multiple regression with up to eight predictors. The estimated minimum sample size was 160 participants, and an additional 25% were recruited to account for potential missing data, yielding a final sample of 200 respondents. Although the study was exploratory, the sample size was calculated based on the secondary analytical objective. This objective was to examine correlations and predictive relationships between motivational, cognitive, and demographic variables. The calculation ensured adequate statistical power for the planned regression analyses.

### 2.3 Variables

The dependent variables in this study were participants’ educational intentions regarding the CNS and PA roles. These variables reflected the level of interest and willingness to pursue studies leading to qualification in each of these emerging advanced clinical pathways.

The independent variables included self‐efficacy, career motivation, intrinsic interest, perceived knowledge of the CNS and PA roles, gender, academic background, and cross‐pathway interest. Self‐efficacy was defined as the perceived personal ability to succeed in advanced clinical education and professional practice. Career motivation referred to the perceived importance of job stability, opportunities for professional advancement, and societal contribution associated with advanced clinical roles. Intrinsic interest represented internal, altruistic motivation and the desire to engage directly in patient care. Perceived knowledge captured participants’ subjective familiarity with the professional scope, responsibilities, and educational requirements of the CNS and PA roles. Gender and academic background (holding or not holding an academic degree) were treated as individual characteristics potentially influencing educational intentions. Cross‐pathway interest referred to participants’ simultaneous interest in one role while expressing curiosity or openness toward the alternative pathway, allowing exploration of overlapping motivational factors.

The control variables included participants’ age, education level, employment status, prior exposure to CNS or PA care (either personally or through family members), and previous consideration of a healthcare career. These factors were recorded as background characteristics that might influence perceptions and intentions regarding advanced clinical education.

### 2.4 Tools

Data were collected using a structured, self‐administered questionnaire composed of sections adapted from previously validated instruments and items identified in the literature on healthcare career choice and advanced clinical roles. The structure of the questionnaire was guided by the Social Cognitive Career Theory (Lent and Brown, 2019). Relevant subscales measuring self‐efficacy, career motivation, and intrinsic interest were adapted from the validated Healthcare Career Choice and Nursing Career Choice scales (Liaw et al. 2017), which have been widely used in nursing and allied health research. Items assessing perceived knowledge and educational intentions toward CNS and PA roles were adapted from prior studies examining professional role awareness and motivation for advanced clinical careers (e.g., Cawley and Hooker 2018; Gullick et al. 2019).

The questionnaire underwent a rigorous translation and cultural adaptation process following standard cross‐cultural validation guidelines (Beaton et al. 2000). This included forward translation from English to Hebrew by two bilingual experts, back translation by an independent translator, and reconciliation by a multidisciplinary expert panel of five academics specializing in nursing education, behavioral sciences, and health policy. The panel reviewed the translated version to ensure content validity, semantic equivalence, and cultural appropriateness. Minor wording adjustments were made to improve clarity and relevance to the Israeli context.

A pilot test with 30 participants from the target population was conducted solely to assess the clarity and comprehensibility of the items and was not used to evaluate reliability or construct validity. Importantly, these pilot participants were not included in the final study sample. The finalized questionnaire consisted of five sections: (1) sociodemographic and background data (age, gender, education level, employment status, and previous exposure to CNS or PA care); (2) perceived knowledge of CNS and PA roles; (3) motivational and cognitive predictors (self‐efficacy, career motivation, and intrinsic interest); (4) educational intentions toward CNS and PA studies; and (5) one open‐ended question for additional comments.

All closed‐ended items were rated on a 5‐point Likert scale (1 = strongly disagree to 5 = strongly agree). Internal consistency reliability for this study was calculated only for descriptive purposes using the final study sample and not as part of new instrument validation. Cronbach's α coefficients ranged from 0.76 to 0.86 across subscales, indicating good internal consistency of the Hebrew version. Because all subscales were adapted from previously validated instruments, construct validity was established in earlier research and not evaluated using the current study population.

### 2.5 Bias

Efforts were made to minimize potential sources of bias at each stage of the study. To reduce selection bias, participants were recruited from diverse demographic and professional backgrounds through open public distribution of the survey link on social media and community platforms, without targeting specific groups. Participation was voluntary and anonymous. To reduce information bias, the questionnaire was self‐administered online with standardized instructions and uniform wording. The instrument combined previously validated and culturally adapted measures to ensure conceptual and linguistic equivalence. To minimize social desirability bias, participants were assured of complete confidentiality and the absence of any identifying information. Responses were collected electronically without researcher involvement to prevent interviewer influence. Finally, measurement bias was addressed through the use of reliability‐tested subscales (Cronbach's α = 0.76–0.86) and expert review to ensure content validity of the Hebrew version.

### 2.6 Procedure

Ethical approval for the study was obtained from the Hillel Yaffe Medical Center Helsinki Committee (Approval No. HY‐0164‐24). An invitation to participate was posted in Facebook groups dedicated to individuals exploring academic study opportunities in Israel. The post included a brief explanation of the study's purpose and a request for volunteers.

Individuals who expressed interest were contacted directly and provided with a personalized questionnaire via Facebook Messenger, WhatsApp, or email, along with a detailed cover letter explaining the study's objectives, confidentiality measures, and the voluntary nature of participation. Completion of the questionnaire required approximately 15 minutes. Data were collected between late 2024 and early 2025. In total, 230 questionnaires were distributed, of which 200 were completed and returned, yielding a response rate of 87%.

### 2.7 Data Analysis

Data were analyzed using IBM SPSS Statistics version 29. Descriptive statistics (means, standard deviations, frequencies, and percentages) were calculated to summarize participants’ sociodemographic characteristics and responses to all study variables.

All quantitative variables derived from the questionnaire were analyzed as continuous data. Each construct, namely, self‐efficacy, career motivation, intrinsic interest, perceived knowledge, and educational intention toward the CNS and PA roles, was represented by a mean score calculated from the relevant Likert‐scale items (range, 1–5). Continuous variables were summarized as M ± SD, and categorical background variables (e.g., gender, education level, employment status, prior exposure to CNS/PA care) were presented as frequencies and percentages. Missing data were minimal (<5%) and were handled using listwise deletion for correlation and regression analyses, ensuring comparable sample sizes across tests.

Bivariate associations among continuous variables were examined using Pearson's correlation coefficient (*r*). The strength of the correlation was interpreted as weak (|*r*| = 0.10–0.29), moderate (|*r*| = 0.30–0.49), or strong (|*r*| ≥ 0.50). Statistical significance was set at *p* < 0.05 (two‐tailed). Two separate multiple linear regression analyses were conducted to identify predictors of educational intention toward the CNS and PA pathways. Each model included the continuous predictor variables corresponding to the theoretical model (self‐efficacy, career motivation, intrinsic interest, and perceived knowledge) and relevant demographic covariates. Analyses were performed using the raw mean values of each scale, consistent with the continuous nature of the data.

## 3 Results

The study included 200 adults who expressed interest in pursuing higher education. The sample was predominantly female (68.5%) and Jewish (88%), with a mean age of 33.1 years (SD = 1.2; range, 18–50, IQR = 27–38). The educational background of participants varied: 51% (*n* = 102) held an academic degree, 18.5% (*n* = 37) had some higher education without a completed degree, and 30.5% (*n* = 61) reported a high school diploma as their highest level of education. Most participants (80%) were employed at the time of the study, and 58% had considered pursuing a career in a healthcare profession (Table 1).

**TABLE 1:** Sociodemographic characteristics of participants (*N* = 200).

Characteristic	Category	%	*n*	Mean (SD)	IQR
Gender	Male	31.5%	63		
	Female	68.5%	137		
Ethnicity	Jewish	88.0%	176		
	Muslim	6.5%	13		
	Christian	2.5%	5		
	No religion	3.0%	6		
Marital status	Single	55.5%	111		
	Married	37.0%	74		
	Divorced	6.5%	13		
	Widowed	1.0%	2		
Educational background	Academic degree	51.0%	102		
	Higher education without a completed degree	18.5%	37		
	High school diploma	30.5%	61		
Employment status	Employed	80.0%	160		
	Unemployed	14.0%	28		
	Seeking employment	6.0%	12		
Considered pursuing a career in a healthcare profession	No	42.0%	84		
	Yes	58.0%	116		
Age	—	—	—	33.1 (1.2)	27–38

Abbreviation: IQR, interquartile range.

A total of 20.5% of participants (*n* = 41) reported personally receiving care from a CNS, and 23.5% (*n* = 47) had received care from a PA. Additionally, 25% (*n* = 50) indicated that a family member had received care from a CNS, while 27.5% (*n* = 55) reported that a family member had been cared for by a PA.

The mean score for interest in pursuing the CNS educational pathway was M = 2.74 (SD = 1.26), while the mean score for interest in pursuing the PA educational pathway was M = 2.48 (SD = 1.08). In total, 26.5% of participants reported interest in pursuing the CNS educational pathway, and 17% expressed interest in the PA educational pathway. When asked whether they would recommend these educational pathways to others, 38% endorsed the CNS pathway, and 30% endorsed the PA pathway.

Intrinsic interest in a healthcare career was notably high, with a mean score of M = 4.25 (SD = 0.55). Specifically, 91.5% of respondents agreed or strongly agreed with the statement “I want to help others,” and 77.5% endorsed “I want to make a difference in someone's life.” Self‐efficacy also scored highly, with a mean of M = 4.12 (SD = 0.63). Notably, 88% of participants agreed or strongly agreed with the item “I believe I can succeed in a healthcare profession.” Career motivation received moderately high ratings, with a mean score of M = 3.89 (SD = 0.58). Among the individual items, 85% of participants agreed with “This profession offers long‐term job stability,” and 78% agreed that “This career provides opportunities for advancement.”

In contrast, perceived knowledge of the CNS and PA roles was relatively limited. The mean score for perceived knowledge of the CNS role was M = 3.10 (SD = 1.02), with only 25.5% of respondents reporting familiarity. For the PA role, the mean was M = 2.95 (SD = 1.08), with 21% indicating familiarity. A substantial proportion of respondents selected “neutral” or “disagree” when asked about their familiarity with the scope of practice for each role, reflecting low levels of subjective awareness (Table 2). Perceived knowledge scores were analyzed as continuous variables to preserve the full range of responses and variability across participants, rather than recoding into dichotomous categories.

**TABLE 2:** Descriptive results of key study variables (*N* = 200).

Variable	No. of Items	Scale range	Mean (SD)	Minimum	Maximum
Self‐efficacy	6	1–5	4.12 (0.63)	2.2	5.0
Career motivation	5	1–5	3.89 (0.58)	2.1	5.0
Intrinsic interest in healthcare	4	1–5	4.25 (0.55)	2.7	5.0
Perceived knowledge of CNS role	3	1–5	3.10 (1.02)	1.0	5.0
Perceived knowledge of PA role	3	1–5	2.95 (1.08)	1.0	5.0
Interest in CNS educational pathway	3	1–5	2.74 (1.26)	1.0	5.0
Interest in PA educational pathway	3	1–5	2.48 (1.08)	1.0	5.0

Correlational analyses revealed several significant associations between key variables, presented below in descending order of strength. Interest in pursuing the CNS educational pathway was most strongly associated with self‐efficacy (*r* = 0.45, *p* < 0.001), followed by career motivation (*r* = 0.32, *p* < 0.001), intrinsic interest in a healthcare career (*r* = 0.264, *p* < 0.001), and age (*r* = 0.228, *p* < 0.01). In contrast, interest in pursuing the PA educational pathway was most strongly correlated with perceived knowledge of the PA role (*r* = 0.357, *p* < 0.001), followed by self‐efficacy (*r* = 0.258, *p* < 0.001), career motivation (*r* = 0.192, *p* < 0.01), and age (*r* = 0.177, *p* < 0.05) (Table 3). Regarding interest in pursuing the CNS educational pathway, women reported significantly higher levels (M = 2.84) compared with men (M = 2.36). Conversely, for the PA educational pathway, men expressed greater interest (M = 2.74) than women (M = 2.39).

**TABLE 3:** Correlations between key variables and interest in pursuing CNS and PA educational pathways (*N* = 200).

Variable	Interest in CNS pathway	Interest in PA pathway
Self‐efficacy	0.45^***^	0.26^***^
Career motivation	0.32^***^	0.19^**^
Intrinsic interest	0.26^***^	—
Perceived knowledge	—	0.36^***^
Age	0.23^**^	0.18^*^

*Note*: Pearson correlation coefficients are presented.

* *p* < 0.05.

** *p* < 0.01.

*** *p* < 0.001 (two‐tailed).

Two separate multiple linear regression analyses were conducted to identify predictors of interest in pursuing the CNS and PA educational pathways (Table 4). Educational intentions toward the CNS and PA roles were measured on 5‐point Likert scales (range, 1–5) and analyzed as continuous outcome variables. This approach allowed for capturing the full variability in participants’ interest levels and maintaining statistical sensitivity, rather than dichotomizing responses into “yes/no” categories.

**TABLE 4:** Predictors of interest in pursuing CNS and PA educational pathways: Results of two multiple linear regression models (*N* = 200).

Predictor	*B* (CNS)	SE (CNS)	β (CNS)	*t* (CNS)	*p* (CNS)	B (PA)	SE (PA)	β (PA)	*t* (PA)	*p* (PA)
Self‐efficacy	0.62	0.08	0.529**	7.75	< 0.001	0.09	0.09	0.088	1.02	0.310
Career motivation	0.31	0.15	0.278*	2.06	0.047	0.07	0.07	0.053	0.98	0.329
Interest in pursuing PA educational pathway	0.29	0.06	0.271***	4.83	< 0.001	—	—	—	—	—
Interest in pursuing CNS educational pathway	—	—	—	—	—	0.34	0.07	0.317***	4.78	< 0.001
Male gender	0.18	0.07	0.157*	2.55	0.011	0.14	0.06	0.144*	2.20	0.030
Academic background	—	—	—	—	—	0.22	0.06	0.213***	3.77	< 0.001
Perceived knowledge						0.09	0.09	0.098	1.02	0.420

*Note*: B = unstandardized coefficient; SE = standard error; β = standardized beta coefficient; *t* = *t* statistic; *p* = significance value. Gender was coded as 0 = female and 1 = male. Dashes (–) indicate variables not applicable to the model or excluded from the final model due to lack of statistical significance.

* *p* < 0.05.

** *p* < 0.01.

*** *p* < 0.001 (two‐tailed).

The first model, predicting interest in pursuing the CNS educational pathway, was statistically significant and accounted for 33.2% of the variance (*R* ^2^ = 0.332, F(5, 194) = 19.25, *p* < 0.001). The strongest predictor was self‐efficacy (β = 0.529, *p* < 0.001), followed by interest in pursuing the PA educational pathway (β = 0.271, *p* < 0.001), and male gender (β = 0.157, *p* = 0.011). Career motivation was also a significant predictor (β = 0.278, *p* = 0.047).

The second model, predicting interest in pursuing the PA educational pathway, explained 25.6% of the variance (*R* ^2^ = 0.256, F(5, 194) = 13.32, *p* < 0.001). Significant predictors included interest in pursuing the CNS educational pathway (β = 0.317, *p* < 0.001), academic background (β = 0.213, *p* < 0.001), and gender (β = 0.144, *p* = 0.030). In this model, self‐efficacy, perceived knowledge, and career motivation were not significant predictors.

## 4 Discussion

This study provides insights into public perceptions, perceived knowledge, and educational intentions regarding two emerging advanced clinical roles in the Israeli healthcare system: CNS and PA. Despite growing national efforts to expand and formalize these roles, findings indicate that public understanding of their scope and function remains limited. Only about a quarter of participants reported perceived knowledge of either role, and personal or familial exposure to CNSs or PAs was similarly low.

As of 2023, approximately 683 CNSs and around 100 certified PAs were practicing in Israel (Ministry of Health 2024; Nursing Administration 2023). The relatively limited availability and visibility of these professionals likely contribute to the observed knowledge gap. This finding aligns with international literature: in countries where CNS and PA roles are still evolving, integration challenges commonly include unclear role definitions, limited public engagement, and underdeveloped training infrastructure (Dickman et al. 2018). These systemic barriers highlight the need for coordinated strategies to raise public awareness and clarify role expectations.

Despite the low levels of perceived knowledge, participants demonstrated neutral to mildly positive attitudes toward both roles. Specifically, 38% indicated they would recommend the CNS pathway, and 26.5% expressed personal interest in pursuing it. For the PA role, 30% reported they would recommend it, and 17% expressed interest in pursuing the PA educational pathway.

This contrast between limited awareness and relatively high endorsement mirrors patterns found in the international literature. For instance, Swainston et al. (2024) reported that UK patients expressed satisfaction with PA care despite limited understanding of the role. Similarly, King et al. (2024) found that patients often confused PAs with physicians but still valued their care experiences.

Findings regarding the CNS role also reflect broader global trends. Tracy et al. (2020) found that, despite institutional ambiguity, CNSs were perceived as highly effective and accessible. In Israel, Ben Natan et al., 2024 reported similar perceptions among patients (Ben Natan et al. 2024) and care teams (Dopelt et al. 2023), emphasizing that role clarity and integration remained ongoing challenges. These results suggest that positive personal interactions can shape public attitudes, even in the absence of comprehensive knowledge.

This study also explored motivational and cognitive predictors of educational intentions. Interest in pursuing the CNS educational pathway was significantly associated with self‐efficacy, career motivation, and intrinsic interest in a healthcare career. These findings align with Social Cognitive Career Theory (Lent and Brown, 2019), which emphasizes the role of perceived capability and internal motivation in career decision‐making.

In contrast, interest in pursuing the PA educational pathway was primarily predicted by perceived knowledge of the PA role and participants’ academic background. These findings imply that external exposure and familiarity, rather than internal drive, currently shape interest in the PA track. Although self‐efficacy and career motivation were not significant predictors in the regression model, they did show modest yet statistically significant correlations in bivariate analyses. This suggests that internal motivational factors may still play a secondary role in shaping PA‐related educational intentions, particularly among individuals who are already aware of the role. This interpretation is supported by previous research emphasizing the importance of role clarity and professional identity in attracting students to emerging health professions (Gullick et al. 2019; Rizzolo et al. 2017). The lack of stronger associations may reflect the PA role's relative novelty and limited formal definition within Israel's healthcare system.

Gender‐based patterns also emerged, with women reporting higher interest in pursuing the CNS educational pathway, while men expressed greater interest in pursuing the PA educational pathway. These trends are consistent with prior studies showing that nursing‐related careers are perceived as relational and aligned with traditional female roles (Begeny et al. 2020; Huang et al. 2024), whereas PA positions were historically viewed as more male‐oriented. However, in recent years, women have come to represent the majority of practicing PAs in many countries, reflecting a shift toward greater gender diversity in the profession (Kozikowski et al. 2024). Such perceptions, even if outdated, may still unconsciously shape career preferences among academically inclined adults.

Finally, the observed positive correlation between interest in pursuing CNS and PA educational pathways suggests a conceptual overlap in how these roles are perceived. This overlap may reflect a broader openness among participants to advanced practice careers in general. Zhao et al. (2024) noted that CNSs and PAs are often viewed as complementary roles, especially within team‐based care models. Promoting these roles in tandem, through integrated awareness campaigns and educational programs, may enhance public recognition, reduce gender role biases, and support workforce development in advanced clinical practice. These findings, drawn from the Israeli context, provide a case example that may inform international strategies aimed at increasing public awareness and engagement with emerging advanced clinical roles.

### 4.1 Limitations

Several limitations should be acknowledged. First, the study employed a convenience sampling method using online recruitment platforms, which may limit the generalizability of the findings. The sample overrepresented Jewish participants and highly educated individuals, which does not reflect the full diversity of the Israeli population. Second, the self‐report nature of the survey may introduce social desirability bias, particularly regarding participants’ expressed intentions and attitudes. Third, the cross‐sectional design prevents any causal inferences about the relationships between variables.

Additionally, the relatively low baseline knowledge among participants about CNS and PA roles may have constrained their ability to provide fully informed responses. This knowledge gap could have influenced how participants interpreted role‐related questions, thus affecting the accuracy of their reported attitudes and intentions. The absence of qualitative data further limits the depth of insight into participants’ motivations and perceptions.

Another limitation of this study concerns the lack of detailed information about participants’ occupational fields. While 80% of participants were employed and 58% had considered a healthcare career, the questionnaire did not specifically identify whether respondents were currently working in healthcare professions. This factor may have influenced the level of awareness and interest reported toward the CNS and PA roles, representing a potential source of unmeasured confounding. Future studies should include occupation type to control for this variable.

Finally, gender was analyzed as a single dichotomous variable (0 = female, 1 = male) to examine potential differences in educational intentions. While gender differences were observed, the study was not designed to explore the sociocultural or contextual factors that might underlie these patterns.

## 5 Conclusion and Recommendations

This study aimed to examine the Israeli public's awareness, attitudes, and educational intentions regarding the roles of CNSs and PAs. The findings revealed limited public knowledge but considerable openness to learning about and engaging with these roles. These results indicate an opportunity to strengthen the integration of CNSs and PAs into the healthcare system through targeted strategies.

To advance this goal, recommendations are offered at multiple levels. From a nursing policy perspective, structured public education campaigns are essential to clarify the professional scope of CNSs and PAs. At the organizational level, enhancing the visibility and recognition of these roles within healthcare teams can help normalize their presence and support professional identity. From a nursing education standpoint, early exposure to advanced practice roles in secondary and higher education settings may foster future recruitment.

These recommendations, grounded in the study's findings, suggest that improved public understanding and strategic investment in education and policy can facilitate the development of a responsive and future‐ready nursing workforce.

## 6 Implications

Nurses in clinical settings play a central role in shaping public perceptions of advanced practice roles. By clearly articulating their professional responsibilities and those of CNSs and PAs during interactions with patients and families, they can foster trust, demystify roles, and enhance care transparency. Additionally, interprofessional collaboration that highlights these roles contributes to stronger team‐based care and public confidence in role legitimacy.

At the policy level, the results support the need for national efforts to raise awareness of CNS and PA roles, especially in addressing workforce shortages and expanding access to care. Nursing policy should promote formal recognition of advanced practice roles, support their integration into strategic workforce planning, and provide incentives for professional training. These steps will help align public perceptions with healthcare needs and contribute to a more sustainable and effective nursing workforce.

## Author Contributions

**Merav Ben Natan**: conceptualization, methodology, writing – original draft. **Yuval Ben Zeev**: data curation, review and editing. **Nicole Bezelanski**: writing – review and editing, supervision.

## Funding

The authors have nothing to report.

## Conflicts of Interest

The authors declare no conflicts of interest.

## Ethics Statement

This study has been approved by the institutional Helsinki Committee (Reference no. HY‐0164‐24).
